# Simultaneous stability and sensitivity in model cortical networks is achieved through anti-correlations between the in- and out-degree of connectivity

**DOI:** 10.3389/fncom.2013.00156

**Published:** 2013-11-07

**Authors:** Juan C. Vasquez, Arthur R. Houweling, Paul Tiesinga

**Affiliations:** ^1^Department of Neuroinformatics, Donders Institute for Brain, Cognition and Behavior, Radboud University NijmegenNijmegen, Netherlands; ^2^Department of Neuroscience, Erasmus Medical CenterRotterdam, Netherlands

**Keywords:** barrel cortex, detection threshold, nanostimulation, degree distribution, computational model, network motifs

## Abstract

Neuronal networks in rodent barrel cortex are characterized by stable low baseline firing rates. However, they are sensitive to the action potentials of single neurons as suggested by recent single-cell stimulation experiments that reported quantifiable behavioral responses in response to short spike trains elicited in single neurons. Hence, these networks are stable against internally generated fluctuations in firing rate but at the same time remain sensitive to similarly-sized externally induced perturbations. We investigated stability and sensitivity in a simple recurrent network of stochastic binary neurons and determined numerically the effects of correlation between the number of afferent (“in-degree”) and efferent (“out-degree”) connections in neurons. The key advance reported in this work is that anti-correlation between in-/out-degree distributions increased the stability of the network in comparison to networks with no correlation or positive correlations, while being able to achieve the same level of sensitivity. The experimental characterization of degree distributions is difficult because all pre-synaptic and post-synaptic neurons have to be identified and counted. We explored whether the statistics of network motifs, which requires the characterization of connections between small subsets of neurons, could be used to detect evidence for degree anti-correlations. We find that the sample frequency of the 3-neuron “ring” motif (1→2→3→1), can be used to detect degree anti-correlation for sub-networks of size 30 using about 50 samples, which is of significance because the necessary measurements are achievable experimentally in the near future. Taken together, we hypothesize that barrel cortex networks exhibit degree anti-correlations and specific network motif statistics.

## Introduction

Rodents can be trained to use their whiskers to detect an object that predicts a reward and respond with licking to obtain this reward (Huber et al., 2012). The neural responses in barrel cortex to whisker stimulation are hypothesized to play an important role in performing this task (Petersen and Crochet, 2013). Animals can also be trained to detect electrical microstimulation (Butovas and Schwarz, 2007; Houweling and Brecht, 2008) or optogenetic stimulation (Huber et al., 2008) of barrel cortex. Microstimulation activates a large number of neurons that are spatially distributed within a few hundred microns around the stimulating electrode (Histed et al., 2009). An important question is how many neurons need to be activated for the subject to reliably detect the stimulation and whether some cell types are more sensitive than others. Answers to these questions may come from nanostimulation experiments in which a single neuron is activated through juxtacellular stimulation (Houweling and Brecht, 2008). These experiments show that adding trains of 10-15 action potentials in a single cortical neuron can indeed be detected, but the reliability of detection is low and reaction times are long compared to microstimulation.

The spontaneous firing rates in the barrel cortex are low, ranging from less than 1 Hz in the superficial layers to a few Hz in the deep layers (de Kock and Sakmann, 2009; Barth and Poulet, 2012), and whisker stimuli typically evoke a single spike (or none) in responsive neurons. The activity in the low firing rate state (LFS) is also stochastic, both in time as well as across cells, but the precise nature of sparse firing is still being quantified (Barth and Poulet, 2012). For a LFS a single spike could represent a significant perturbation, potentially yielding 28 additional spikes in postsynaptic neurons (London et al., 2010). The network state therefore needs to be stable against small fluctuations that may be amplified through recurrent connectivity. At the same time the aforementioned experiments show that the network is sensitive to small perturbations that are externally generated. Sensitivity and stability are connected and can in general not be optimized at the same time, as the increase in one causes a decrease in the other. Furthermore, stable LFS, in the sense of asynchronous and irregular activity, is difficult to achieve (Kumar et al., 2008).

We use two insights to find the optimal trade-off between stability and sensitivity. First, the external and internal generated firing rate fluctuations may have different statistics. The external perturbation is a train of action potentials [e.g., of 200 ms duration (Houweling and Brecht, 2008)] in a single neuron, thus correlated in time, whereas the internal fluctuations are likely to be of shorter duration and involve a more diverse set of neurons. Second, network structure may be such that these fluctuations have different stability properties (possibly through learning). Our guiding hypothesis is that simultaneous stability and sensitivity are achieved through an anti-correlation between the in- and out-degree of synaptic connectivity between neurons in barrel cortex. Thus, neurons with a low number of synaptic inputs have a high number of synaptic outputs and neurons with a high number of inputs have a low number of outputs. We further hypothesize that such an anti-correlation leads to a distribution of synaptic connectivity motifs that is different than for a random network (Milo et al., 2002). Experiments show that barrel cortical circuits have a motif distribution that is different from random (Song et al., 2005; Perin et al., 2011), whereas theoretical studies show that networks with non-random motif distribution have different synchronization properties (Roxin, 2011; Zhao et al., 2011; Litwin-Kumar and Doiron, 2012) (LaMar and Smith, 2010) and can emerge through synaptic plasticity during reward-based learning (Bourjaily and Miller, 2011a,b). Our work is the first that focuses on the effect on network dynamics of correlations between the in- and out-degree of the same neuron, rather than between in- and/or out-degrees of different neurons, which is referred to as assortativity (Newman, 2010).

Here we test these hypotheses in simplified networks of neurons. In order to focus on the effect of network structure, rather than the full dynamics of spiking neurons, we model neurons as binary units. The inputs to the binary units are determined through a connection matrix with a pre-specified degree distribution generated by a configuration model (Newman, 2010). We first describe how the networks are constructed and then determine (1) their stability in terms of the maximal coupling constant for which the LFS is still stable and (2) their sensitivity to single-cell perturbations using a receiver operating characteristic (ROC) analysis. Finally, we address the issue of how to detect evidence for anti-correlations in the degree distribution experimentally on the basis of sampling sub-networks.

Taken together, we find that anti-correlated networks are more stable than equivalent correlated and uncorrelated networks, but can still reach the same level of sensitivity, which represents a key theoretical advance in terms of a hypothesis for the experimentally observed sensitivity and stability of neuronal networks in the rodent barrel cortex. Furthermore, the hypothesis is of experimental significance, because our analysis shows that correlations in the degree distribution can be detected using sub-networks of sizes that are experimentally accessible in the near future.

## Methods

### Network dynamics

The model network was composed of *N* binary excitatory neurons, whose state at time *t* is given by *x*_*i*_(t), a *N*-dimensional vector with ones for neurons that are active and zeros for ones that are not, here *i* is the index of the neuron. The new state *x*_*i*_(t + 1) is obtained in two steps. First, the probability ν_*i*,*t* + 1_ of a neuron being active is calculated using Equation (1). Second, for each neuron the firing probability is compared to a random number that is uniformly distributed between 0 and 1. The neuron is set to 1 when the random number is less than or equal to the probability value

(1) $$v_{i,\;t\;+\;1}=\frac{1}{1+\exp\text{​}\left(h_{0}-\frac{J}{Np_{c}}\sum_{j}w_{ij}x_{j,t}\right)}$$

The probability has a sigmoidal form, with the exponent consisting of a constant term *h*_0_, which sets the probability of firing in the absence of inputs from other neurons, and a coupling term representing the network input. The coupling term contains the adjacency matrix *w*_*ij*_, whose construction is described below, and in which *w*_*ij*_ = 1 if there is an input from neuron *j* to neuron *i* and *w*_*ij*_ = 0 otherwise. The overall probability of a connection is *p*_*c*_. Hence the sum across rows of the adjacency matrix is on average *Np*_*c*_ and we normalize the coupling term by *J*/*Np*_*c*_ so that *J* then represents the overall coupling strength. The network activity is calculated in time bins that we consider to be 10 ms. The network has a high firing rate state (HFS), in which each neuron is active on each time step, to which the network will converge when enough neurons are active on a previous time step. We are primarily interested in the LFS, in which each neuron fires only in a fraction of the time bins, corresponding to a firing rate of approximately 1 Hz (Barth and Poulet, 2012). Alternatively, in a given time bin, only a fraction of neurons are active.

The network activity is represented by the mean probability of firing of a neuron during a time bin and is calculated as the total number of spikes divided by the number of neurons. When normalized by the bin width, it represents the mean firing rate of a network neuron in spikes per second (Hz).

### Network connectivity

Our goal is to determine whether correlations in the in- and out-degree distribution are beneficial in that they increase sensitivity and/or stability relative to uncorrelated networks. Hence, we need a control network without degree correlations. Although the standard random network, Erdos-Renyi (ER) (Newman, 2010), does not have correlations in the degree distribution and is easy to generate samples of, it is not appropriate as a control because it has a sharp degree distribution (see below) and we instead need large variance degree distributions.

For ER networks with a connection probability *p*, the degree distribution (for both out- as well as in-degree) is given by a binomial distribution

(2) $$p(k)=\left(\begin{array}{c} N-1\\ k \end{array}\right)p^{k}{\left(1-p\right)}^{N-1-k}$$

which has a mean of (*N* − 1)*p* and a variance of (*N* − 1)*p*(1 − *p*), which in the limit of large *N* converges to Gaussian distribution with a ratio of the standard deviation over the mean of

(3) $$\sqrt{\frac{1-p}{N}}$$

**Figure 1:**
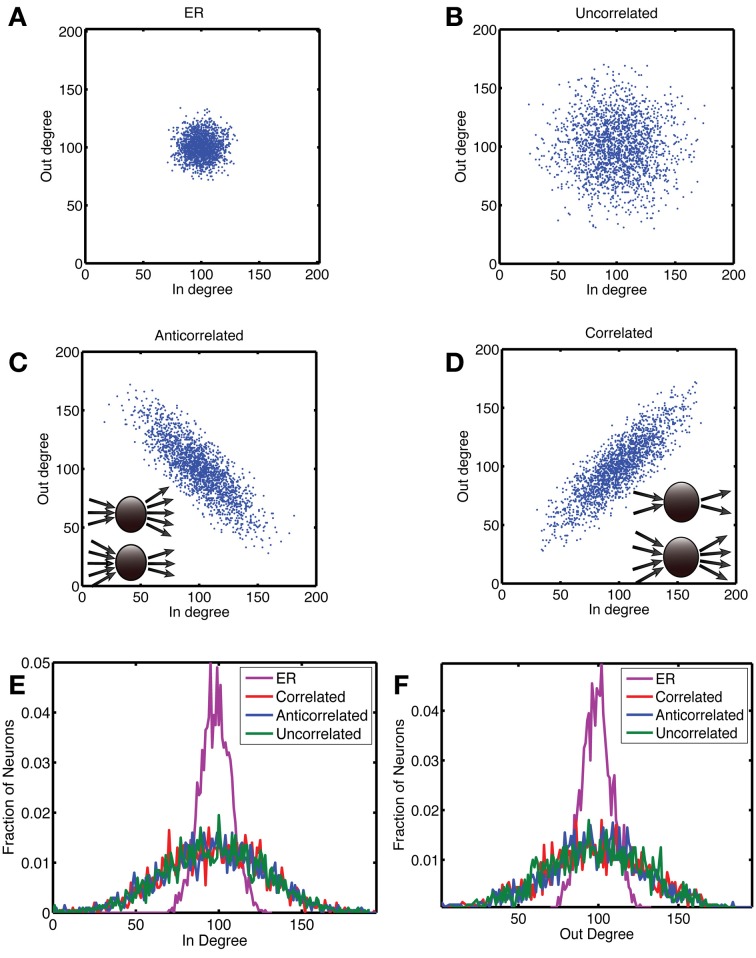
**Construction of networks with a correlation between out- and in-degree.** In panels **(A)** to **(D)**, we show scatterplots of the out-degree vs. in-degree, whereas the corresponding marginal distributions are shown for **(E)** the in-degree and **(F)** the out-degree. We considered four types of networks, each with *N* = 2000 neurons and a connection probability of *p*_*c*_ = 0.05. **(A)** The Erdos-Renyi (ER) network in which each connection is chosen at random with a probability *p*_*c*_ = 0.05, for which there is no correlation [ρ = 0.0034 (standard deviation: 0.018)] and the relative variance of in- and out-degree across neurons is small for large networks. In order to examine networks with a higher variance of degree values, we first generated a degree distribution in the form of a truncated, bivariate Gaussian. In **(B)** the covariance matrix was diagonal, with equal variance for the out- and in-degrees, which yielded uncorrelated in- and out-degrees [ρ = 0.0010 (0.019)]. To generate correlations we started from a covariance matrix with unequal variances and rotated it by 45 degrees anticlockwise to obtain **(C)** anti-correlated [ρ = 0.821 (0.0085)] and by 45 degrees clockwise to obtain **(D)** correlated degree distributions [ρ = 0.821 (0.0085)]. In the anti-correlated case, nodes with a high out-degree had a low in-degree and vice versa, whereas in the correlated case, nodes with a high out-degree also had a high in-degree, as illustrated by the insets in **(C)** and **(D)**, respectively. **(E,F)** The networks were constructed so that the marginal distributions for the correlated (red), anti-correlated (blue) and uncorrelated (green) case were the same. The ER network (purple) had much tighter marginal distributions.

This means that the distribution becomes very tight for large network sizes (Figures 1A,E,F).

Hence we generated networks from a truncated bivariate Gaussian for the joint in- and out-degree distribution as explained below (Figures 1B–F). We start from a bivariate Gaussian with a diagonal covariance matrix given by

(4) $$p(x,y)=\frac{1}{\sqrt{4\pi^{2}}\sigma_{x}\sigma_{y}}\exp\text{​}\left(-\frac{{\left(x-\mu \right)}^{2}}{2\sigma_{x}^{2}}-\frac{{\left(y-\mu \right)}^{2}}{2\sigma_{y}^{2}}\right)$$

which is rotated across 45 degrees clockwise or anticlockwise to obtain a distribution with positive and negative correlations, respectively. The resulting distribution is truncated below at 1 because the degree cannot be negative and we exclude the case of zero (since a zero degree neuron would not be considered part of the network) and above at twice the mean degree to make the distribution symmetric. The resulting distribution is normalized to make the integral over the positive quadrant equal to one. The short axis is represented by σ_x_ and the long axis is represented by σ_*y*_. The mean degree μ was equal to *Np*_*c*_, with a network size *N* = 2000 and connection probability *p*_*c*_ = 0.05 (Holmgren et al., 2003) this yields μ = 100. The long axis was σ_*y*_ = μ/3. The term dispersion refers to the ratio σ_*x*_/σ_*y*_, which was set to 0.3 for the standard parameter set.

Correlated degree distributions were obtained by sampling for each neuron *i*, the in- and out-degree from the above bivariate Gaussian, *d*^in^_*i*_ and *d*^out^_*i*_. The simplest method for generating a realization of the corresponding network is the configuration method (Newman, 2010). A list with *d*^out^_*i*_ stubs with value *i*, is made and concatenated into a list *s*^out^_*k*_. Likewise, a list with *d*^in^_*i*_ stubs with value *i*, is made and concatenated into a list *s*^in^_*k*_ and randomly permuted. From these two lists, pairs are picked from the same position, i.e., the *k*th stub on the out-list is matched to the *k*th stub on the in-list to make the connection *s*^out^_*k*_ to *s*^in^_*k*_. This algorithm produces networks with two artifacts, there could be self-connections *s*^out^_*k*_ = *s*^in^_*k*_, and a given connection could be sampled twice (or more), *s*^out^_*k*_ = *s*^out^_*l*_ and *s*^in^_*k*_ = *s*^in^_*l*_. For sparse networks the likelihood of self-edges is small (0.05%), but the probability for multi-edges was larger, around 2.7%. For the cases in which there were multi- or self-edges, we removed the corresponding links.

### Network stability

Cortical networks with a low firing rate need to be stable in the sense that stochastic fluctuations should not lead to large increases in the firing rate that could be detected as a stimulation, resulting in a false positive. We characterized the network stability in three ways.

First, we simulated the network and determined the mean firing rate, averaged across neurons and across time bins, as a function of the coupling strength *J* for various levels of background activity *h*_0_. To determine both the maximal stability and tease apart the contribution of neuronal heterogeneity and stochasticity to instability, we performed the simulations according to a number of different schemes. We considered the mean field limit, in which the network is taken to be so large that each neuron received the same number of inputs and that the resulting mean firing rate of each neuron was the same. Equation 1 reduces in that case to

(5) $$v_{t+1}=\frac{1}{1+\exp(h_{0}-Jv_{t})}$$

yielding the following equation for the fixed points

(6) $$v=\frac{1}{1+\exp(h_{0}-Jv)}$$

which correspond to the roots of the function

(7) $$f(x)=x-\frac{1}{1+\exp(h_{0}-Jx)}$$

and can be obtained by iterating the fixed point equation Equation 6 or using Matlab's root finder fzero. The background field h_0_ determines the baseline firing rate *r*_0_, which is the rate obtained in the absence of coupling, *J* = 0:

(8) $$r_{0}=\frac{1}{\Delta t}\frac{1}{1+\exp(h_{0})}$$

where we have divided by the bin size Δt to obtain a firing rate in Hz.

There is always a high firing rate solution for sufficiently high coupling strength *J*, because when all neurons are active on a given time step, they will also all be active on the next time step. There can also be a low firing rate solution which depends on the coupling strength and the baseline firing rate. The coupling strength *J*_*c*_ at a given baseline firing rate below which the LFS exist is the upper limit of stability. The stochastic dynamics generates fluctuations, which could push the network away from the LFS, whereas a degree distribution with a large variance would cause a dispersion in the mean firing rate across neurons. These effects are characterized by performing the full simulations without stochasticity to determine the effect of firing rate dispersion,

(9) $$v_{i,t+1}=\frac{1}{1+\exp\left(h_{0}-\frac{J}{Np_{c}}\sum_{j}w_{ij}v_{j,t}\right)}$$

and the stochastic version in Equation (1) to determine the effect of fluctuations.

Second, in the latter case, the state (LFS vs. HFS) reached is not deterministic, because a network can have a firing rate that fluctuates around the LFS or veers off to the HFS due to a somewhat larger fluctuation. We therefore performed the simulation multiple times and recorded how often (on what fraction of the trials) the network ended up at the HFS state as a function of the coupling constant. In this case we defined *J*_*c*_ to be the value of the coupling constant at which 50% of the states converged to the HFS within 400 time steps. The initial condition of the network was obtained by making a random set of neurons active in such a way that on average it had the same number of active neurons as expected based on the firing rate in the mean-field limit.

Third, when fluctuations stay in the basin of attraction (BOA) of the LFS, the network will not diverge, which means that the above fraction is an indirect measure of the BOA. We also determined a more direct measure by starting networks from different initial conditions, each with a different number of active neurons, and determining which fraction of trials goes to the HFS within 400 time steps. These initial states are characterized by the effective number *N*_eff_ of active neurons as is explained in the Results section and represented in Equation 11.

### Network sensitivity

The sensitivity to a perturbation in experiment is tested in the model by activating a few selected neurons for a fixed duration. The stimulation was characterized by the number *n*_*p*_ of neurons stimulated (typically *n*_*p*_ = 8), the number of time bins, *T*_stim_, the stimulation lasted (typically *T*_stim_ = 6) and the mean out-degree of the stimulated neurons represented by *N*_eff_. For a fair comparison between different networks we randomly picked the stimulated neurons from the network and repeated the stimulation for 50 different realizations of the network. In order to estimate the effect of out-degree on the detection of the stimulation, we also ordered neurons based on their out-degree, with the highest out-degree first. This ordered set was divided into ten groups of equal size. We then randomly selected the stimulated neurons from a specific group and compared how the network response depended on which group was being stimulated.

### ROC analysis

The ROC is obtained by picking a threshold and determining how often a firing rate response from the unstimulated network exceeds this threshold: the fraction of false positives. In addition, it is determined how often the firing rate of the stimulated network exceeds this threshold, this is the fraction of true positives. The ROC curve is traced out by plotting the true positives vs. the false positives for each possible threshold. When the distributions are exactly the same, the number of true positives equals the number of false positives, hence the ROC is the diagonal with an area under the curve (AUC) of 0.5. The deviation of the ROC curves from the diagonal, or equivalently deviation of the AUC from 0.5, is a measure for how different the distributions are and maps for Gaussian distributions on to d′, which is the difference in means of the distributions divided by the standard deviation (Kingdom and Prins, 2010). This also means that one can determine how many trials are needed to detect, given a particular ROC value, a difference between stimulation trials and unstimulated responses. The errors in the ROC curve and AUC value were determined by resampling of the simulated trials. Typically *N*_*r*_ = 2000 resamplings were used.

### Fuzzy clustering and perceptron analysis

Fuzzy c-means (FCM) was used to cluster data points, such as a vector of network firing rates in consecutive time bins, or the motif distribution for a particular realization of a network, into groups with similar properties. FCM can be understood by first considering *K*-means clustering. In a *K*-means clustering, a number of clusters is chosen and the objects to be clustered are assigned on a random basis to each of the potential clusters (Duda et al., 2001). The name of the algorithm derives from the convention that the number of clusters is denoted by *K*. Using these assignments, the mean of each cluster is found. Then, using these means, objects are re-assigned to each cluster based on which cluster center they are closest to. This process repeats until the cluster centers have converged onto stable values or a maximum iteration count is reached. This type of clustering minimizes the sum of the squared distances of the clustered objects from their cluster means. FCM functions in the same way, but rather than belonging to any particular cluster, each object *i* is assigned a set of normalized probabilities *u*_*ij*_ of belonging to cluster *j* (Bezdek, 1981). This is equivalent to minimizing a non-linear objective function of the distances of the objects from the cluster centers, characterized by the “fuzzifier” parameter, which is set to two. After the algorithm converges each data point is assigned to the cluster to which it is most likely to belong (maximizing the *u*_*ij*_ with respect to the cluster index *j*). A more complete description is given in (Fellous et al., 2004).

The perceptron algorithm is a method to classify responses x of the network (Duda et al., 2001). Here the vector *x* = (*r*_*t*_, *r*_*t*+1_) represents either a point in the firing rate return map or it represents the binary activity for each neuron during a particular time bin. The algorithm tries to find a weight vector *w* such that the sign *w*^*T*^x is positive when *x* belongs to group 1 (stimulated network) and negative when it belongs to group 2 (unstimulated).

### Analysing motif count distributions

To investigate whether we could use motif statistics (restricting ourselves to 3-node motifs) of smaller parts of the complete network to distinguish between networks with different degree correlations, we generated *N*_*r*_ = 1000 realizations of each network type: correlated, anti-correlated and uncorrelated. We used smaller networks, *N* = 200, because these networks are adequate to represent sub-network statistics of size *N*_sub_ up to 200. We used standard parameters, *p*_*c*_ = 0.05, now yielding μ = 10 and σ_*y*_ = μ/3 = 3.33 and σ_*x*_ = 0.3σ_*y*_ = 1.0 for the smaller network. From each realization we sample sub-networks of *N*_sub_ from 4 to 24 in steps of 4 and from 30 to 200 in steps of 10. For each (sub) network we count the number of 3-node motifs using the explicit formulas given in Table III of Itzkovitz et al. (2003). Each motif is labeled by a number according to the convention also found in Itzkovitz et al. (2003). The counts in an ER network vary with powers of the expected number of edges per node *k* and network size *N*, λ*N*^3^(*k*/*N*)^*e*^, where λ is a factor representing the symmetry of the pattern [see Table III in Itzkovitz et al. (2003)] and *e* is the number of edges in the pattern, which defines the complexity of the motif.

As a first step in the analysis we determined the mean and standard deviation of the motif count across the *N*_*r*_ realizations. To reduce the size of statistical fluctuations we also pooled motif counts by averaging them across *N*_*av*_ realizations. We either split the original *N*_*r*_ realizations into *N*_*r*_/*N*_*av*_ groups, yielding a reduced number of data points or we randomly sampled with replacement *N*_*r*_*N*_*av*_ samples from the original *N*_*r*_ samples to keep the same number of pooled motif counts. The count distribution was often not Gaussian, which meant we could not use the *t*-test for the difference in mean count over the standard deviation. Hence, we utilized a ROC analysis. In order to obtain error estimates we created *N*_*b*_ = 20 different sets of *N*_*r*_ = 500 realizations, each of which were obtained by randomly sampling with replacement from amongst the *N*_*r*_ = 1000 original realizations.

We also wanted to determine whether incorporating counts of pairs of motifs would improve the ability to distinguish between networks with different degree correlations. We considered each realization, drawn from one or the other group of networks, as a two-dimensional data point and used FCM to find two clusters. FCM outputs the confidence (or probability) that the data point belongs to cluster 1. This value can be used as part of an ROC procedure. For a given threshold, the true positive corresponds the fraction of data points belonging to group 1 for which the confidence exceeds the threshold, whereas the false positive corresponds to the fraction exceeding threshold that belongs to the second group. We applied this procedure for each possible pair of motifs and for each sub-network size.

## Results

### Anti-correlated networks are most stable in the zero-noise case

**Figure 2:**
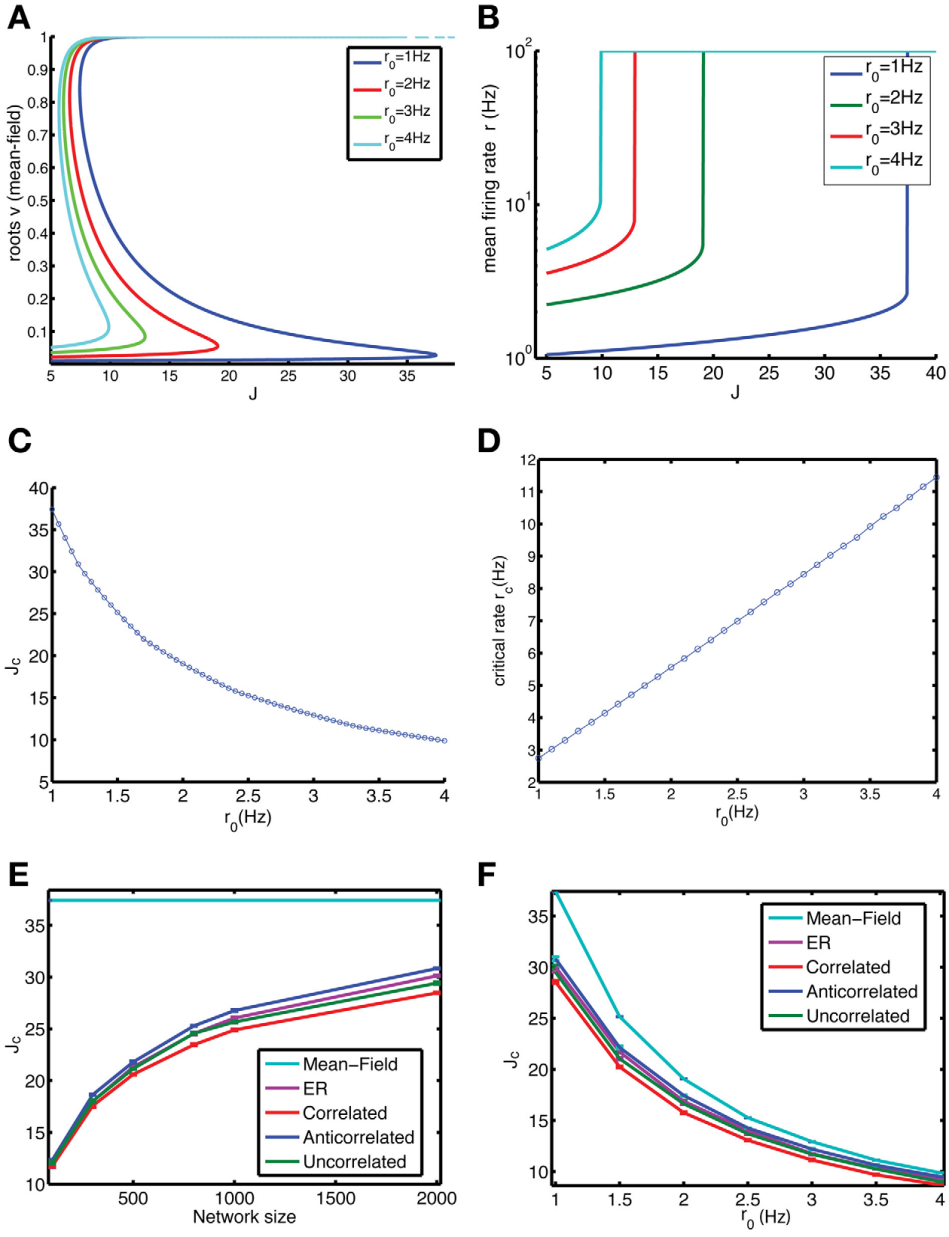
**Anti-correlations in the degree distribution improve the stability of the low firing rate state (LFS).** We compared the stability of finite-size networks with different degree correlation structure by iterating Equation 9 (which is Equation 1 without taking into account stochastic spiking). **(A)** The mean-field solution, corresponding to an infinite-size network, is simulated by assuming that the firing rate of each neuron is equal, yielding Equation 6, of which all roots are shown in the graph. **(B)** Mean firing rate r vs. coupling constant J in the mean-field limit for different values for the baseline firing rate *r*_0_. When the LFS loses stability, the only remaining solution is the HFS. As a result the plotted firing rate suddenly jumps to the maximum possible rate of 100 Hz (corresponding to 1 spike per bin). **(C)** The range of stable coupling constants, which are between 0 and *J*_*c*_, decreases with increasing baseline firing rate. **(D)** The firing rate *r*_*c*_ of the LFS just before it turns unstable increases linearly with *r*_0_. **(E)** The stability of the LFS depends on system size and approaches the mean-field limit (cyan) gradually as network size *N* increases (baseline rate *r*_0_ = 1 Hz). The anti-correlated network (blue) is always more stable than the ER (purple), correlated (red), and uncorrelated (green) networks. **(F)** The difference between the mean field *J*_*c*_ and that of the finite-size networks decreases with baseline firing rate (network size *N* = 2000).

The mean-field limit, corresponding to an infinite network, is studied by considering the dynamics of a network where each neuron has the same firing rate, each neuron has the same number of synaptic inputs, i.e., in-degree, and there is no stochasticity. In this case the dynamical equations reduce to a self-consistent equation for the average firing rate *v* (Equation 6 in Methods), which is solved according to the fixed point method. There are typically two stable solutions, one corresponding the HFS, in which the neuron is constantly firing (firing probability *v* = 1 or close to one) and one corresponding to the LFS at much lower rates, together with one unstable solution in between (Figure 2A). For high enough coupling constants only the HFS solution remains. We studied this by starting from an initial value of *v*_*t*_ near zero and then iterating Equation 5 until convergence, if there is a LFS, it will converge to the LFS and if there is no LFS it will converge to the HFS, resulting in a sudden jump in firing rate as a function of *J* (Figure 2B). The coupling strength for which this jump occurs is denoted by *J*_*c*_ and depends on the baseline firing rate *r*_0_ (defined in Equation 8, Figures 2B,C). The higher *r*_0_ the less stable the network is. The firing rate of the LFS for *J* values just before it becomes unstable, referred to as *r*_*c*_, is the maximum firing rate that the network can sustain, which varies approximately linear with the baseline firing rate (Figure 2D).

The effect of network size is studied by iterating Equation 9 for a vector of firing rate values, which ignores the effects of noise that are present in the full equations, Equation 1. In these finite size systems, the LFS is less stable, as reflected in the *J*_*c*_ values that are much below the mean-field limit (Figure 2E). There also is a difference between networks depending on their degree correlations, with the anti-correlated network being more stable than the ER network, correlated and uncorrelated networks. These differences become more pronounced for larger networks (Figure 2E). The difference also depends on the baseline firing rate, with the anti-correlated network again being the most stable (Figure 2F).

### Anti-correlated networks are more stable against fluctuations

**Figure 3:**
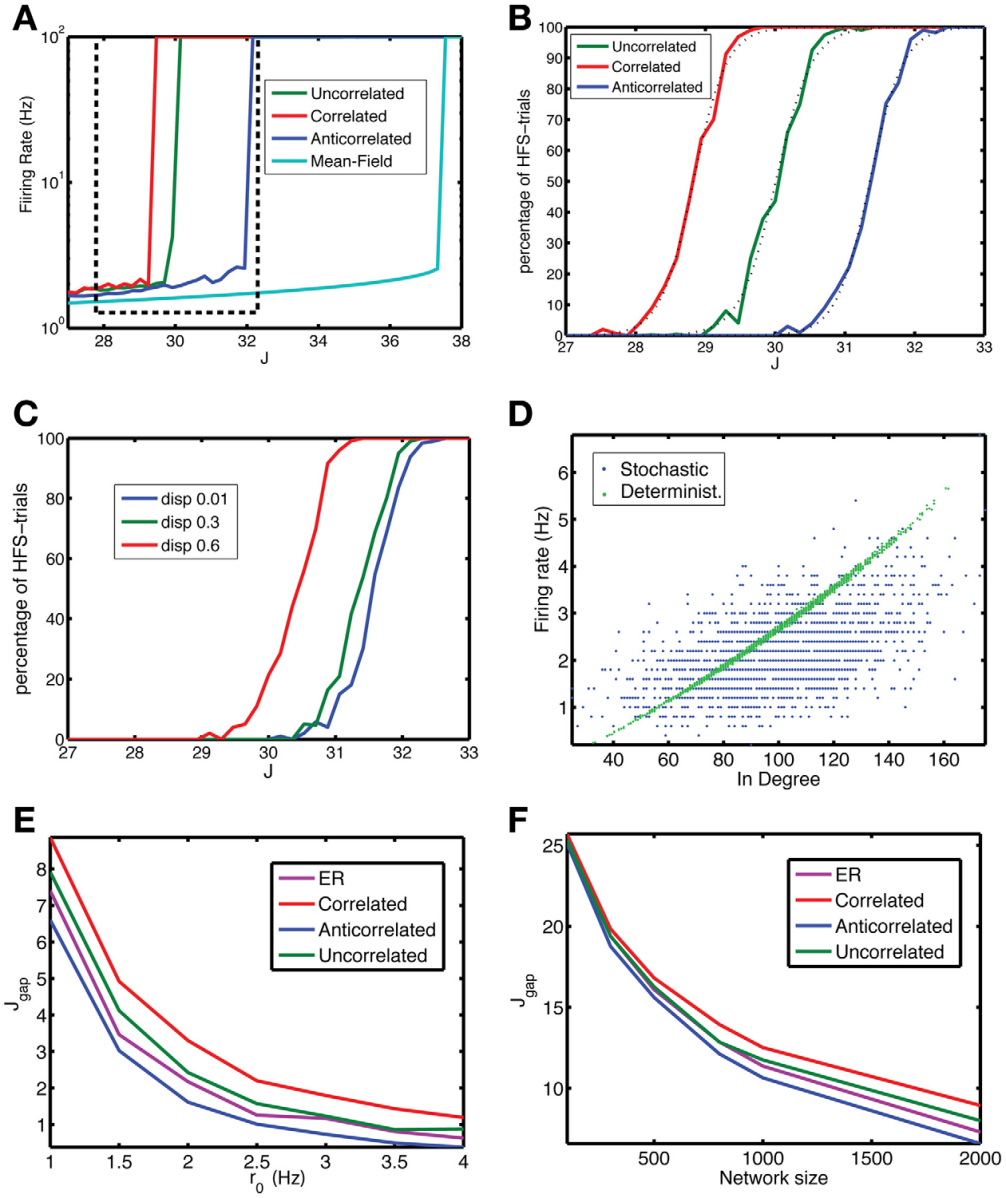
**The anti-correlated network is more stable against fluctuations. (A)** The firing rate vs. coupling strength for the mean-field solution (cyan) and networks with uncorrelated (green), correlated (red) or anti-correlated (blue) degree distributions (*r*_0_ = 1 Hz, *N* = 2000). The anti-correlated degree distribution leads to the most stable network. The dashed box approximately indicates the interval of coupling strengths highlighted in panels **(B)** and **(C)**. **(B)** Despite the existence of a stable LFS for a particular coupling strength, fluctuations in network activity may perturb the network away from it and the network ends up in the co-existing stable HFS state. The fraction of states that end up in the HFS state is close to zero far below *J*_*c*_ and increases to unity above *J*_*c*_. The LFS state is more stable for the anti-correlated (blue) network than for the uncorrelated network (green), which in turn is more stable than the correlated network (red). The dashed lines are fits to the sigmoidal function in Equation 10. **(C)** The stability depends on the strength of the correlation. When the width (dispersion) corresponding to the small axis in the bivariate Gaussian degree distribution is increased, which means lower correlation, the stability is reduced. Data are for an anti-correlated network. **(D)** A neuron's firing rate is correlated with its in-degree, but the degree of correlation is reduced to 0.519 (0.014) due to jitter in this relation for Equation 1 (blue dots) from 0.997 (0.002) for Equation 9 (green dots). Data for anti-correlated network, *J* = 30.96. **(E,F)** The degree of stability can be qualified by *J*_gap_, the distance of the *J*_*c*_ for the finite-size network from that for the mean-field network, shorter distances meaning more stable networks. *J*_gap_ decreases with the **(E)** baseline firing rate *r*_0_ and with **(F)** network size. In both panels the anti-correlated network (blue line) corresponds to the lowest curve indicating higher stability compared to ER (purple), uncorrelated (green) and correlated (red), an advantage that increases with network size. The network had *N* = 2000 neurons, for each coupling strength *N*_*t*_ = 100 simulations were performed, with a length of 500 time steps, of which the first 100 were discarded as a transient.

The dynamics of binary networks is stochastic because on each time step the expected firing rate is translated into a binary value. Hence, the firing rate, either averaged across network neurons during one time bin, or of one neuron averaged over a few time bins, will fluctuate. These fluctuations will alter the stability because these fluctuations could drive the network out of the BOA of the LFS toward that of the HFS state. The firing rate in the LFS state vs. coupling constant curve for the stochastic network (Figure 3A) looks similar to that for the zero noise case (not shown), but the fraction of trials on which the HFS state is reached displays a sigmoidal behavior (Figure 3B): with some networks switching to the HFS state close to, but below the critical coupling constant *J*_*c*_, whereas most of the networks go to HFS for coupling constants above *J*_*c*_. In between there is a transition point where an equal number of networks go to the LFS and HFS state. The anti-correlated state is more stable, because this transition point lies to the right of the transition point for the other networks (Figure 3B). We have fitted the probability to the following expression,

(10) $$\text{p}(\text{J})=1/(1+\text{exp}(-\left(\text{J}-{\text{J}}_{\text{h}}\right)/\sigma_{\text{J}})$$

where *J*_*h*_ is the transition point and σ_*J*_ represents the sharpness of the transition. The transition for correlated and anti-correlated networks is sharper than for uncorrelated networks, as indicated by the σ_*J*_ = 0.424 and 0.420, compared to 0.391, respectively, with *R*^2^ values (fraction of explained variance) all approximately 0.999.

The in- and out-degrees are drawn from a bivariate Gaussian, which has a long axis, in the direction of the correlation, and a short axis perpendicular to that direction (Equation 4, Methods). Increasing the standard deviation along the short axis, termed dispersion, reduces the degree of correlation. In addition, it makes the anti-correlated network less stable (Figure 3C).

The stability properties of the finite-size networks are different from that in the mean-field limit (Figure 2), because the firing rate of a neuron depends on the number of inputs (in-degree), which varies across neurons in the network (Figure 3D, green dots). The correlation between the neuron's firing rate and its in-degree is almost perfect for the non-stochastic network, with squared Pearson correlation *R*^2^ = 0.997 (0.002), but becomes jittered due to the stochastic spiking resulting in a squared Pearson correlation of 0.519 (0.014) (Figure 3D, blue dots).

The mean-field limit represents the highest level of stability, because both finite-size and noise effects reduce it. The reduction in stability can be captured into *J*_gap_, which is the mean-field critical coupling minus the critical coupling value for the noisy, finite-size network. The smaller *J*_gap_ is, the more stable the system is. The gap decreases both with baseline firing rate (Figure 3E) and network size (Figure 3F). As the network size increases, the comparative stability advantage of anti-correlated networks increases.

The stability against fluctuations can be analyzed differently. Non-linear dynamical systems are characterized in terms of the basin of attraction (BOA). Consider a simple one-dimensional system with two stable fixed points (and an unstable one in between) (Strogatz, 1994). Depending on the initial condition of the one state variable, the system will converge to one or the other fixed point. The catchment area of the first fixed point, the range of initial conditions that converge toward it, is the BOA. There is a well-defined boundary between the two BOAs. Our goal is to characterize this boundary between LFS and HFS for the binary networks studied here, which is complicated because of the high dimensionality of the state space and the stochasticity, which means that a given initial condition near the boundary could converge to a LFS or HFS depending on the role of the dice. The first issue means we have to find a more effective and compact description of the initial state. Our initial choice was to use the number *N*_*a*_ of active neurons in the initial condition. However, when the *N*_*a*_ highest out-degree neurons are active, the network is more likely to converge to the HFS than when the *N*_*a*_ lowest out-degree neurons are active, even though the initial state has an equal number of active neurons. Hence, we used the so called effective number of active neurons, where each neuron's contribution is weighted by their out-degree:

(11) $$N_{\text{eff}}=N\frac{\sum_{i\;\in \text{ active}}d_{i}^{\text{out}}}{\sum_{i}d_{i}^{\text{out}}}$$

**Figure 4:**
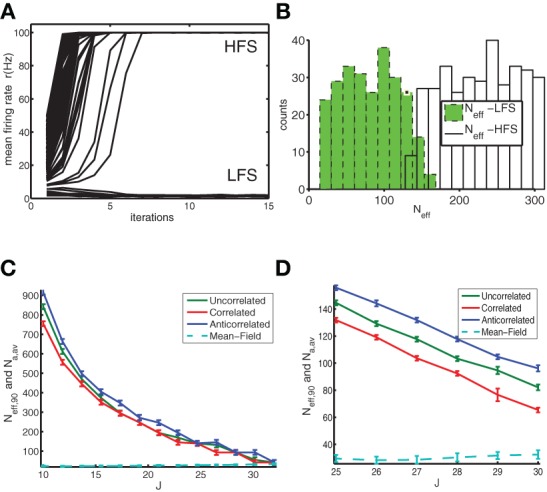
**The basin of attraction of the LFS is larger for anti-correlated networks indicating enhanced stability against fluctuations. (A)** Simulations were started from initial states with a different number *N*_*a*_ of active neurons, which is translated into an *N*_eff_ value (see text) to allow for a fair comparison of initial conditions. We show the firing rate as a function of time (in units of iterations). For low *N*_*a*_ the anti-correlated network converged to the LFS, whereas for high *N*_*a*_ runs it converged to the HFS. **(B)** This was reflected in the histogram where green filled bars indicate the number of states with a particular *N*_eff_ that converged to the LFS and the open bars indicate the number of states that converged to the HFS. Data for anti-correlated network with *J* = 25. **(C)** *N*_eff,90_ as a function of coupling constant *J* for uncorrelated (green), correlated (red) and anti-correlated (blue) networks together with the number *N*_*a,av*_ of active neurons corresponding to the firing rate of the mean-field solution (cyan dashed line) as a reference. **(D)** Close-up of panel **(C)**. The data were obtained from a network of *N* = 2000 neurons, with a baseline rate of 1 Hz. For each coupling strength, and, each network type we used *N*_*r*_ = 1000 initial conditions and averaged across 4 realizations of the network.

We started the simulations from a random initial state, characterized by a specific number of active neurons (range: between 0 and 200), and repeated this procedure enough times (*N*_*r*_ = 4000) to ensure sufficient coverage across the relevant *N*_eff_ values. For each *N*_eff_ value so sampled, a fraction converged to the LFS and the remainder went to the HFS state (Figure 4A). For small *N*_eff_ most states converge to LFS and for *N*_eff_ larger than a transition value *N*_eff,90_ most converge to the HFS (Figure 4B). We choose as transition value the lowest *N*_eff_ value for which 90% or more states went to the HFS. The transition value *N*_eff,90_ decreases with coupling strength *J* (Figures 4C,D) until its value comes close to the number of active neurons represented by the average firing rate of the mean-field network, at which point stability is lost. This is because the BOA of the LFS shrinks to zero and most initial conditions go to the HFS. The anti-correlated network is more stable because it can sustain initial states with a higher number of active neurons and still return to the LFS as compared to other networks. Furthermore, for the anti-correlated networks the BOA is finite for larger values of the coupling constant compared to other networks. Overall, when a sufficient number of neurons are active in the initial condition, both the effective and unnormalized number of active neurons yield similar results for the size of the BOA (not shown).

### The sensitivity of the network can be characterized using ROC analysis

During spontaneous (unstimulated) activity in the network, the firing rate will fluctuate from time bin to time bin, which can be considered random draws from a distribution. When the network is stimulated, the average firing rate will be altered, trivially because of the activated neurons, but non-trivially through the downstream effect of this stimulation on the other neurons. The stimulation is characterized by the number of cells *n*_*p*_ stimulated (and their out-degree, see below) and the duration of the stimulation *T*_stim_. We used *n*_*p*_ = 8 and *T*_stim_ = 6. Its effect on the network can be detected when there is a systematic difference between the network states, quantified, for instance, in terms of the mean firing rate of the overall activity. An ROC analysis quantifies how different the distribution of firing rate is between the stimulated and unstimulated networks and how easy it is to detect this difference and can thus be compared to measured behavioral responses. In all of the following analyses we exclude the stimulated cells themselves. One reason is that the decision process would be based on downstream neurons, hence we should detect the difference in the downstream population.

**Figure 5:**
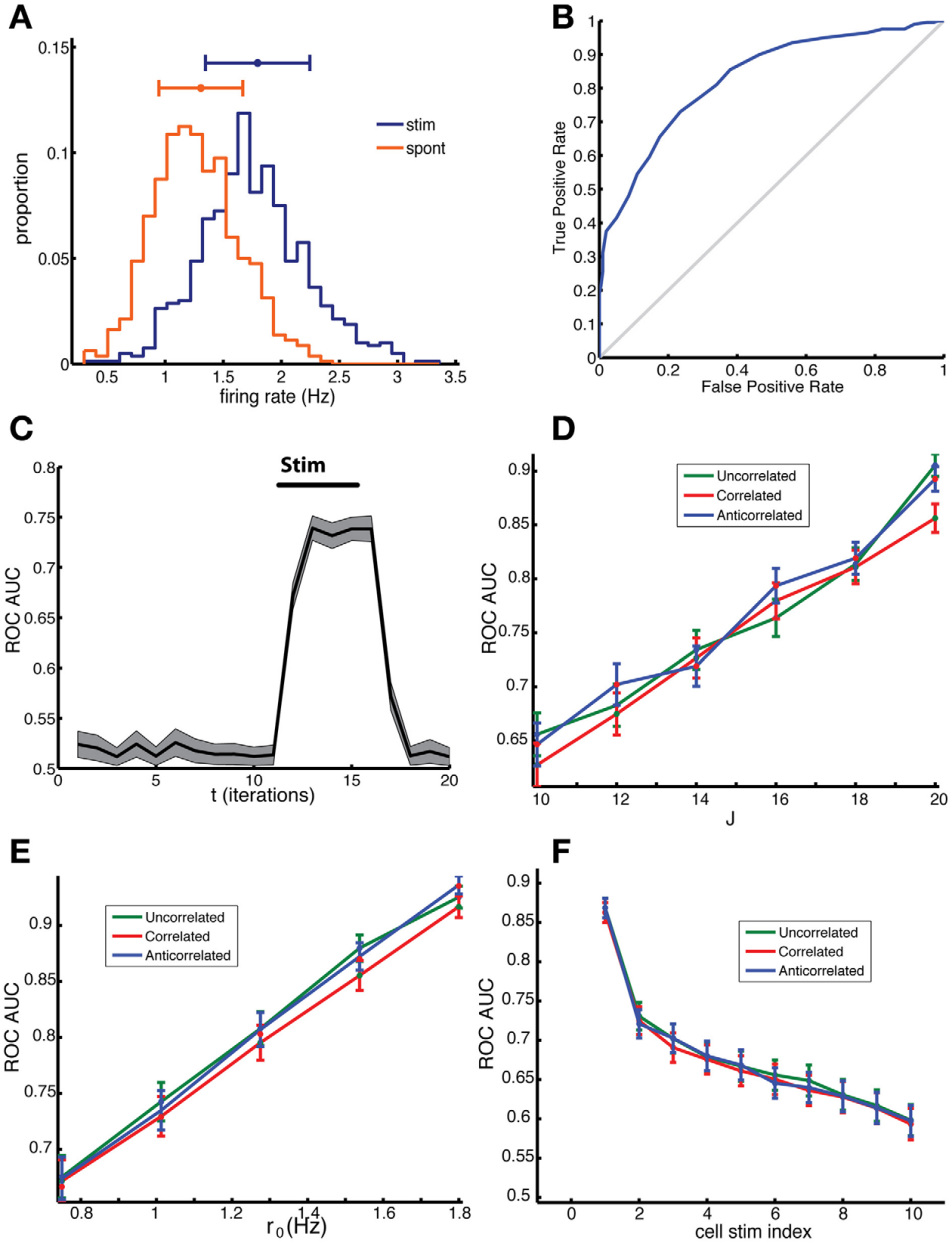
**Network sensitivity, when evaluated using an ROC analysis, depends only on the mean out-degree of the stimulated neurons and not on the degree correlations. (A)** Distribution of firing rate across cells in a 10 ms time bin for spontaneous activity (red) and for the stimulated network (blue), in which 8 random cells were stimulated. Note that the stimulated cells were not included in this ROC analysis and we used the binary responses *x*_*i*_(*t*) to determine the firing rate. **(B)** The corresponding ROC curve (blue) quantifies the difference between the distributions, relative to the diagonal (gray), which represents distributions that cannot be distinguished. **(C)** The area under the curve (AUC) for the ROC curves calculated for different time bins. The AUC before stimulation was close to 0.5 because the distributions were the same apart from fluctuations due to sampling. After the stimulation, which started at *t* = 10 and ended at *t* = 15, the AUC rose to around 0.75. **(D)** The AUC increases with increasing coupling constant and **(E)** with increasing baseline firing rate. **(F)** The AUC depended on the mean out-degree of the stimulated neurons. Neurons were divided into ten groups according to their out-degree, with the first group having the highest out-degree. The group index is indicated on the x-axis. The results in **(D–F)** were not significantly different for correlated (red), anti-correlated (blue) or uncorrelated (green) networks, *t*-test, *p* = 0.4479, 0.6279, 0.7421, respectively. The network was comprised of *N* = 2000 neurons, of which *n*_*p*_ = 8 neurons were stimulated for the duration of *T*_stim_ = 6 time units starting on the 10^th^ bin. In panel **(A–C)** results for an uncorrelated network are shown. Parameters: **(A–C,F)** *J* = 18, *r*_0_ = 1; for **(D)** *r*_0_ = 1 and **(E)** *J* = 20.

The histogram of the simulated firing rates was shifted relative to that of the unstimulated network (Figure 5A). In Figure 5B, the ROC curve corresponding to the empirical distributions in panel a is shown. The evaluation of the corresponding AUC, as a function of time is shown in panel c. Before the stimulation at *t* = 10, the statistics of both networks are the same, yielding an AUC of close to 0.5, whereas after stimulation the AUC rises to the 0.75. The ability to detect a stimulation increases with the strength of the coupling constant (Figure 5D). This can be simply understood because a higher *J* increases the impact of presynaptic activity on the neuron's firing rate, hence it also increases the effect of stimulation. There is no difference in sensitivity due to the correlation structure of the network as long as neurons with similar out-degrees are stimulated, because the sensitivity only depends on the out-degree. The AUC also increases with baseline firing rate of the network (Figure 5E), which indicates that network state changes, such as those occurring during arousal or with attention in which the overall firing rate increases, could improve task performance. Also for this behavior there was no difference between networks with the different type of degree correlations. The derivative of the mean firing rate *r* with respect to *J* increases with baseline firing rate *r*_0_, suggesting that the effect of a stimulation on the network firing rate increases with *r*_0_, which is indeed borne out by the simulation results in Figure 5E.

Stimulus detection depended on which cells were stimulated, with their average out-degree being the most important factor. We chose *n*_*p*_ neurons to be stimulated randomly from 10 different groups with different mean out-degree, which were generated as follows. First all neurons were ordered according to their out-degree, with the highest out-degree neurons coming first, and then divided into ten equally-sized groups, labeled 1 to 10. Multiple stimulation trials were done with *n*_*p*_ neurons picked from one of the groups from which the group AUC was determined. The AUC for each group was then plotted as a function of the group label (Figure 5F). The AUC values for the first group were much higher than for the next groups demonstrating clearly that the group with the highest mean out-degree also had the highest AUC.

Taken together, these simulations show that the correlations in the degree distribution do not directly affect network sensitivity to stimulation. Rather, this sensitivity is determined by the out-degree of the stimulated neurons. Networks can display a higher sensitivity if they have a larger variability in the out-degree distribution and those cells with the highest out-degree are being stimulated. ER networks have a low variance in the out-degree, and will therefore have a reduced sensitivity compared to the networks here, compare the AUC of the first group to that of the fifth group which represents neurons with an out-degree closest to the mean.

**Figure 6:**
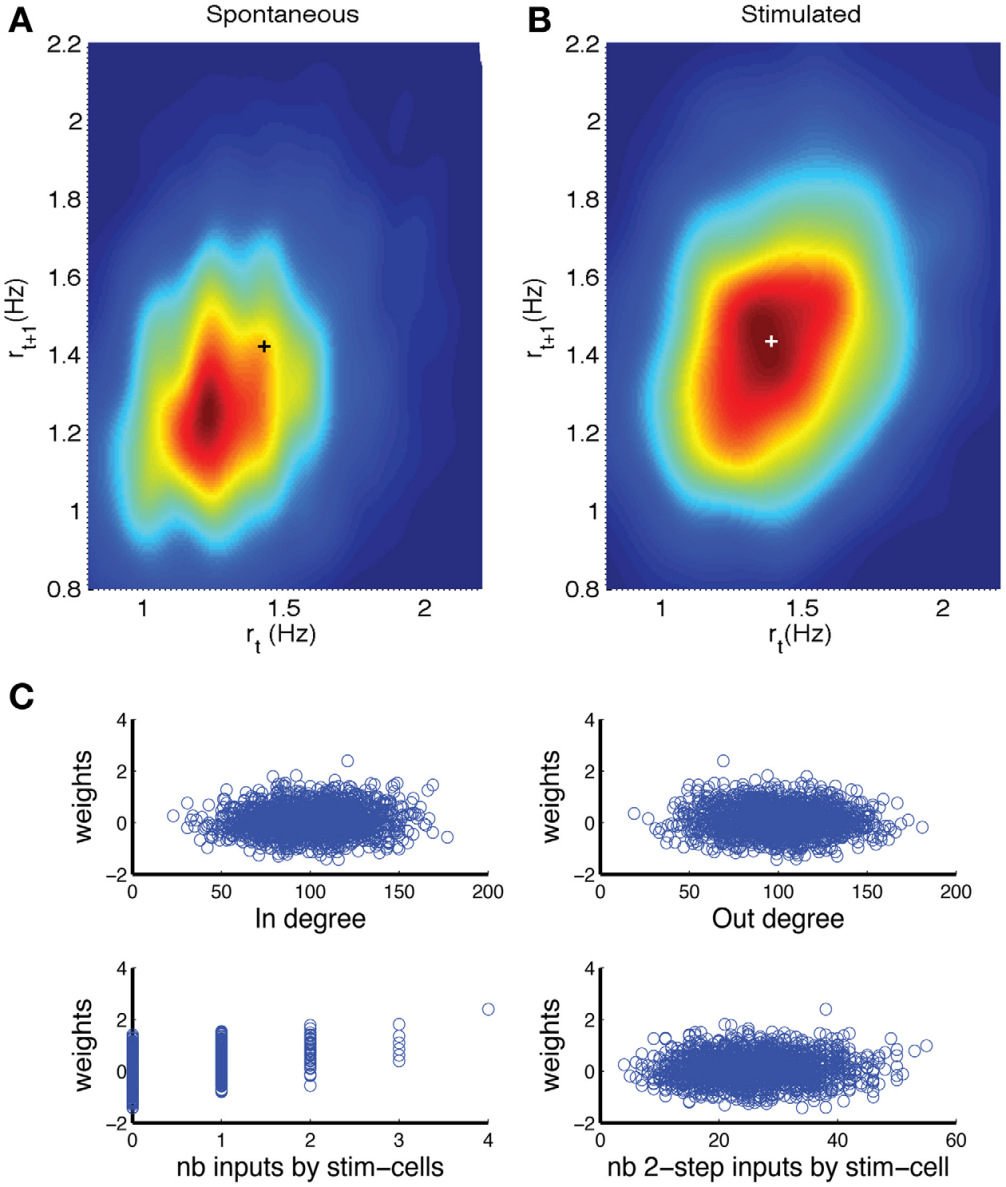
**Detection can be improved by including past activity and weighting neurons depending on how many inputs they receive from directly stimulated neurons. (A,B)** Density representation of the firing rate return map, wherein the probability of obtaining consecutive rate values (r_*t*_, r_*t*+1_) is represented by a color scale, with red indicating the highest probability and blue indicating a near zero probability. The results are shown for **(A)** spontaneous activity and **(B)** stimulated activity. The plusses indicate the location of the peak in panel **(B)**. **(C)** Analysis of factors that contribute to a neuron's weight in detection decision that is outputted by the perceptron procedure. There was significant but small correlation between weight and (top left) in-degree or (top right) out-degree. There was a correlation between the weight and the number of direct inputs from stimulated neurons (bottom left), but only a weak correlation with the number of inputs from cells that received direct input from the stimulated cells (bottom right). The network was comprised of *N* = 2000 neurons, coupling constant *J* = 18, baseline firing rate *r*_0_ = 1 Hz. In the stimulated network, *n*_*p*_ = 8 neurons were stimulated for a duration of *T*_stim_ = 6 time bins.

The fluctuations in firing rate during spontaneous activity are expected to have different temporal correlations compared to those in the stimulated network, as an increase due to an external stimulation is going to persist across the time bins during which the stimulation takes place. Hence, the detection rate could improve by taking into account (spatio) temporal correlations. The first step is to consider the correlation in network firing rate *r* between two consecutive time bins. When *r*_*t*+1_ is plotted vs. *r*_*t*_ a return map would be obtained. However, because the firing rate values are restricted to *x*/(*N*Δ*t*), where *x* is an integer between 0 and *N*, and *N* the network size, the return map would have a non-informative appearance. Hence, we made a density representation, by replacing each sample by a two-dimensional Gaussian (kernel density estimate) with a standard deviation (bandwidth) optimally estimated from data following the Silverman's rule of thumb (Silverman, 1986). The hot spot in the return map density obtained for stimulated networks (Figure 6B, plus sign) is shifted along the diagonal in the positive direction (i.e., higher rates) in comparison to the return map for spontaneous activity (Figure 6A).

We determined whether such a two-dimensional representation would improve the detection rate. An equal number of samples from spontaneous activity and from stimulated activity were provided to a fuzzy clustering method (FCM) routine in Matlab in order to find two clusters (Fellous et al., 2004). The FCM returns for each data point *i* the probability *u*_*ij*_ that it belongs to cluster *j*. As the sum of probabilities needs to be unity, for two possible clusters we only need to consider *u*_*i*1_. We thus obtain a distribution of *u*_*i*1_ values for data points from the spontaneous activity and a distribution for data points from the stimulated network. The difference between these distributions is a measure for how well stimulation can be detected and can thus be subjected to a ROC analysis. In this ROC analysis the *u*_*i*1_ values are treated in exactly the same way as the firing rates used to obtain the results in Figure 5. The resulting AUC values were 5% higher than based on the distribution of firing rates in one bin (*t*-test, *p* = 0).

In the firing-rate based detection procedure, each neuron (except the directly stimulated ones) carries equal weight. The cells that are not directly connected to the stimulated neurons would display firing rate fluctuations that are unrelated to the stimulation, hence act as noise that reduces probability of detection. The signal to noise of the firing rate fluctuations could be improved by weighing those neurons less. To explore this hypothesis we applied a perceptron procedure (see Methods) to learn the optimal weights for classifying the network state vectors (Duda et al., 2001). An equal number of network states for spontaneous activity and for stimulated networks were supplied to the perceptron routine together with the corresponding class labels. The output was a weight for each neuron. As before the activity of the directly stimulated neurons was not included in this analysis. To determine what features contributed to the weight we plotted the weight vs. feature value in a scatter plot and calculated the corresponding Pearson correlation. There was a small, but significant correlation between the weight and the in-degree (Figure 6C, top left, correlation 0.073 ± 0.03, *p* = 0.0011) and with the out-degree (Figure 6C, top right, correlation−0.052 ± 0.027, *p* = 0.018). There was a strong correlation between the weight and the number of direct inputs the neuron received from stimulated neurons (Figure 6C, bottom left, correlation 0.410 ± 0.15, *p* = 0.0). The number of indirect inputs from stimulated neurons was less relevant (Figure 6C, bottom right, correlation 0.072 ± 0.032, *p* = 0.012). We calculated this by determining the number of inputs from cells that received direct inputs.

Taken together, these analyses show that our estimates for the detection of stimulation based on overall firing rate are underestimates and can be improved by taking into account network history and by selecting which neurons to listen to. The latter of which may be achieved through synaptic plasticity and the appropriate learning rules.

### Detecting anti-correlation in the degree distribution with limited data

The results here establish that anti-correlation between in- and out-degrees results in more stable, but equally sensitive networks, compared to networks without correlations between in- and out-degree, or positive correlations between them. Hence, learning to detect a stimulation could proceed by altering the correlation between in- and out-degree. To demonstrate such a learning effect, the in- and out-degree of a number of neurons needs to be sampled. Classical tracing techniques are not appropriate because they involve the connections to or from multiple nearby neurons (Lanciego and Wouterlood, 2011). For instance, when the retrograde tracer horseradish peroxidase is injected, it is absorbed by multiple axon terminals and transported to their respective cell bodies. These axon terminals do not necessarily synapse on one and the same neuron near the injection site. Hence, the data cannot be used to determine the in-degree of a neuron near the injection site.

New viral-based techniques could help, because they work by infecting a few cells in the neighborhood where the virus is injected (Wickersham et al., 2007; Osakada et al., 2011). The virus will then retrogradely label the cells presynaptic to these cells by crossing one synapse and one synapse only. In the presynaptic cells the infection stops because the virus misses the proteins necessary to cross another synapse. The challenge with this method is to infect only one cell, with both an anterogradely and retrogradely crossing virus.

Currently, the gold standard is to simultaneously record multiple cells *in vitro* and assess connections by inducing action potentials in one neuron at a time and recording the post-synaptic responses in the other cells. The current record is 12 cells recorded simultaneously (Song et al., 2005; Perin et al., 2011). This means that the anti-correlation in the degree distribution will have to be assessed indirectly, by sampling from sub-networks.

Motifs represent patterns in the connectivity that occur more often than expected if the connections were made random (Milo et al., 2002). For instance, consider a network for which the average probability of a connection is *p*. For two neurons, if these connections are made randomly, the probability of having no connection is (1 − *p*)^2^, for having one connection 2*p*(1 − *p*) and for having a bidirectional connection *p*^2^. When it is found that bidirectional connections occur significantly more than the expected *p*^2^ then there is additional, non-random, structure in the network (Song et al., 2005). Motifs most often refer to triplets of neurons and the patterns of connectivity between them that occur more often than expected in a random network (Milo et al., 2002). A motif distribution is the number of times each motif occurs in a network and a motif is considered present when it occurs more often than in a control network. Motif distributions are affected by many network properties such as, for instance, the degree distribution. The networks studied here, even when uncorrelated, have a different degree distribution than the ER network, which means that ER random networks are not a good control. Hence, we have to numerically generate the control distributions rather than having access to the analytical expression for the expected rate of each motif. In addition, in experimental settings we do not have access to the whole network from which to determine the motif distribution, we have to do with sub-networks. These sub-networks do not come from the same network, rather they come from networks sampled from an ensemble of networks with similar properties. To obtain estimates for how to observe evidence for anti-correlation in the degree distribution we need to deal with each of these issues.

The overall goal is to distinguish between pairs of networks with anti-correlated, uncorrelated and correlated degree distribution with the same marginal distribution for in- and out-degree.

**Figure 7:**
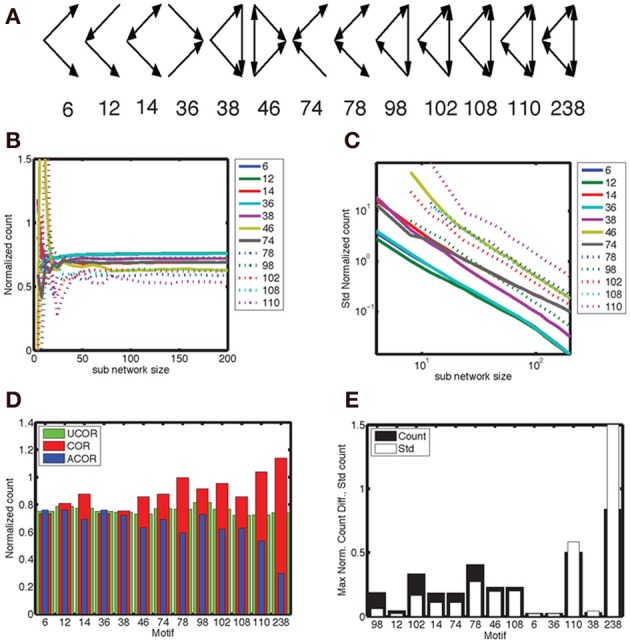
**Motif 98 is the most sensitive to degree correlations. (A)** There are 13 motifs that involve 3 connected nodes. Below the graphical representation we plot the numbering used here, which follows Itzkovitz et al. (2003). The expected number of motifs depends on network size, hence we normalize the count by *N*^3^(*k*/*N*)^*e*^, with *N* the number of nodes, *k* the expected number of edges per node and *e* the number of edges in the motif. In addition, we include a numerical factor representing the equivalent permutations [listed in Table 3 in Itzkovitz et al. (2003)]. **(B)** The normalized counts, averaged across a thousand realizations, converge to constant values for sub-networks larger than 50–100 nodes, with the precise value depending on the complexity of the motif involved. **(C)** The standard deviation of the normalized counts fall off as *N*^−3/2^. We illustrate the results for the anti-correlated network, which are typical for the correlated and uncorrelated network also. In addition, we omitted motif 238 because it occurs at such a low probability that it makes the statistics noisy. **(D)** The normalized counts for each motif for the (red) correlated, (blue) anti-correlated and (green) uncorrelated networks. We used the counts for the full network, rather than sub-networks. Network size in panel **(D)** and **(E)** was *N* = 200. We used a bivariate Gaussian degree distribution with a mean number of nodes equal to 10, a standard deviation along the long axis of σ_*y*_ = 3.33 and along the short axis of σ_*x*_ = 1.0. **(E)** The maximum difference in mean count between all three possible comparisons (black bars), relative to the mean standard deviation of these counts across the three network types. The motifs are ordered on the count over standard deviation ratio, starting with the largest. According to this analysis motif 98 should be used to best distinguish between different network correlation structures.

We considered 13 different motifs that consisted of three connected neurons and gave each motif a numerical label as shown in Figure 7A. We determined the number of motifs in each realization of a network with correlated, anti-correlated or uncorrelated degree distribution and took the average. This was done for the full network (here reduced to *N* = 200) as well as for sub-networks (size *N*_sub_). The complexity of a motif corresponds to the number of edges in the pattern, ranging from 2 to 6, which determines how often it is counted in a network. We normalized the counts such that they took values on the order of unity in order to better compare them across motifs. The mean count as a function of *N*_sub_ converged to a constant for network sizes between 50–100 neurons (Figure 7B), with more complex motifs requiring larger *N*_sub_. The width of the count distribution, quantified as the standard deviation, decreased with *N*_sub_ as the-3/2 power (Figure 7C). Hence, for large enough networks the differences in mean counts across network type can be detected with certainty. This power law behavior is consistent with the results for a Binomial process with probability *p* and on the order of *n* ~ N^3^_sub_ trials, for which the mean is *np* and the variance is *np*(1 − *p*). In that case the normalized mean is *p*, and its variance (*1-p*)*/n* (see also Equations 2, 3), leading to a standard deviation varying as *n*^−1/2^ = *N*^−3/2^_sub_.

We are looking for motifs whose counts are different between the analyzed network types. In Figure 7D we show the count as a function of motif for the three network types, with the highest difference occurring for the complex motifs 110 and 238. However, the counts for these motifs, which have the largest number of edges, are characterized by a large standard deviation. When we plot the motifs in an order based on the ratio of count difference over standard deviation, motif 98 comes up as winner instead (Figure 7E). Figure 7D shows that there are fewer motif 98 in anti-correlated networks compared to correlated networks. This can be understood intuitively by noting that in “ring” motif 98 each neuron has the same number of inputs as outputs, namely 1, which is more representative for correlated networks (Figure 1D, inset) than for anti-correlated networks (Figure 1C, inset). Furthermore, this means there is a lower probability of closing the ring, because in an anti-correlated network a neuron with many inputs has fewer outputs to get to the next neuron in the ring.

**Figure 8:**
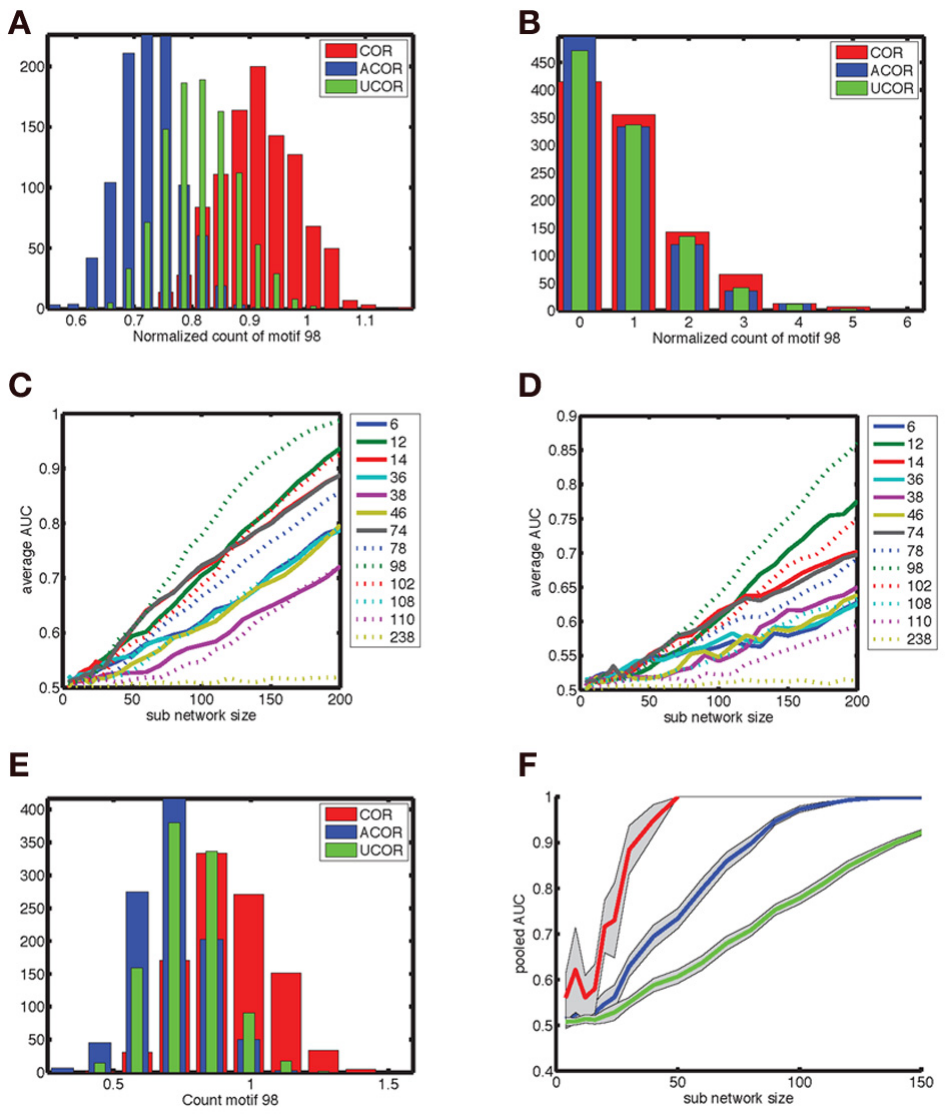
**Degree correlations can be distinguished by pooling fifty measurements of networks at least thirty neurons in size. (A)** Motif count varies across network realizations, but degree correlations can be distinguished when the corresponding distribution show little overlap. We show the distribution of the normalized counts of motif 98 for (red) correlated, (blue) anti-correlated, and (green) uncorrelated networks with 200 neurons. **(B)** For smaller sub-networks (*N*_sub_ = 30), the distributions overlap. Furthermore, these distributions are not Gaussian as they are skewed because counts are always positive. Hence, a more general procedure, such as the ROC analysis needs to be used instead of looking at the differences in mean count relative to the standard deviation. **(C,D)** The area under the ROC curve (AUC) as a function of sub-network size *N*_sub_ for the comparison **(C)** between correlated and anti-correlated networks and **(D)** between anti-correlated and uncorrelated networks. Each motif is labeled with a line style and color as indicated in the legend. Motif 98 is most sensitive in both cases (as well as for the correlated vs. uncorrelated comparison that is not shown). It is more difficult to distinguish an anti-correlated network from an uncorrelated one than to distinguish it from a correlated network. The average AUC values were determined based on the AUC value for each of twenty different motif distributions of 500 network realizations, which were sampled randomly with replacement out of 1000 realizations. **(E)** The motif distribution for *N*_sub_ = 30 can be pooled across *N*_*av*_ = 50 network realizations in order to shrink the width of the distribution, so that the differences in mean counts become clearer [(compare to panel **(B)]**. **(F)** The AUC for larger *N*_*av*_ values reaches unity (distributions are perfectly distinguishable) for smaller sub-network sizes. We show (green) no pooling, (blue) pooling across *N*_*av*_ = 5 realizations and (red) pooling across *N*_*av*_ = 50 realizations. The AUC goes from 0.7 to 1.0 between *N*_sub_ = 30 and 50 when pooled across *N*_*av*_ = 50 realizations, indicating that networks of size 30 can be used to determine degree correlation structure.

The count distributions are not Gaussian for small sub-networks. Figure 8A shows the count distribution for motif 98 for networks with *N* = 200. Each network gives rise to a symmetric appearing distribution, with the peak at a different location depending on the network type. The distribution for the anti-correlated and correlated network were farthest apart, with that of the uncorrelated distribution situated in the middle. For *N*_sub_ = 30 (Figure 8B), the corresponding distributions fell on top of each other and are asymmetric because the counts are always positive. To compare the distributions we therefore performed an ROC analysis. As expected based on the reduced overlap between distributions, the AUC increases with sub-network size, and motif 98 comes out on top with the highest AUC (Figure 8C). Furthermore, given the lower overlap between the anti-correlated and correlated distribution (Figure 8A), the AUC values for the comparison between anti-correlated and correlated network is higher (Figure 8C) than for either the comparison between anti-correlated and uncorrelated (Figure 8D) or correlated with uncorrelated (not shown).

In experiments only relatively small networks can be mapped, up to 12 cells using paired recordings and a few tens to hundreds using population calcium imaging. For these numbers the degree correlations cannot be reliably distinguished based on a single measurement. We therefore pooled measurements to see if this improved discriminability for more experimentally accessible smaller sub-networks. This procedure (pooling motif counts across *N*_*av*_ = 50 network realizations) indeed reduced overlap between distributions (Figure 8E, compare to Figure 8B). The more motif counts were pooled, the higher the AUC was (Figure 8F). Furthermore, the value of unity, corresponding to perfect discriminability is reached for smaller sub-network sizes. For *N*_*av*_ = 50, *N*_sub_ = 30 networks are perfectly discriminable and the AUC transitions from values just above 0.7 to unity between *N*_*av*_ = 30 and 50 (Figure 8F). Taken together, sub-networks of a few tens of neurons could be used to test our hypothesis experimentally.

**Figure 9:**
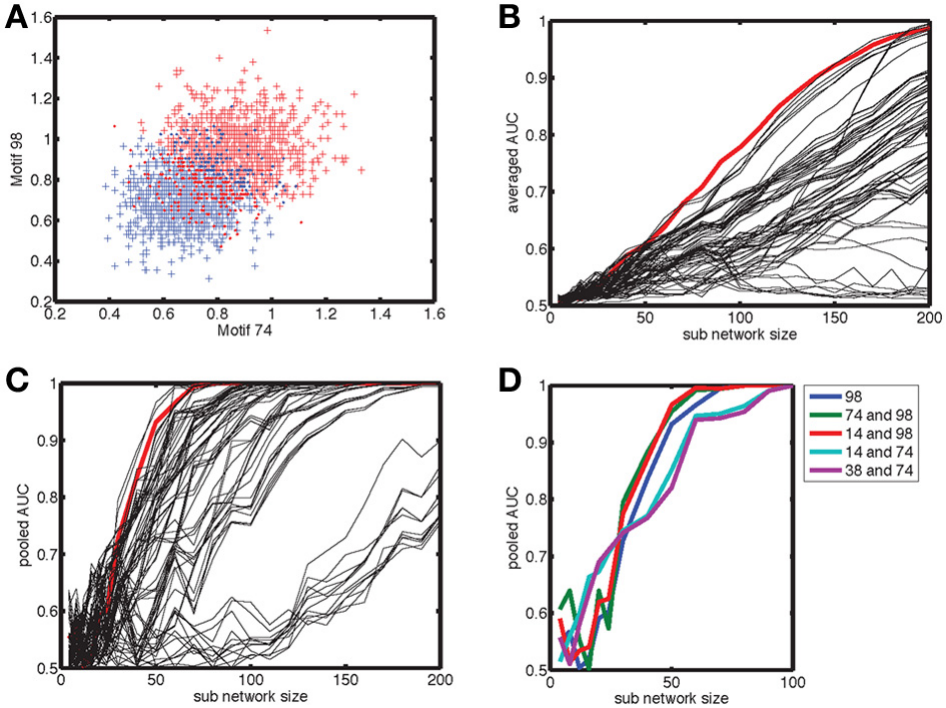
**The gain in discriminability by using the joint distribution of motif counts rather than the marginal distribution is limited. (A)** The outcome of the FCM procedure to find two clusters. Red points indicate counts obtained from a correlated network and blue points are those for the anti-correlated network. The plusses indicate points correctly classified by FCM and the dots represent incorrectly classified network realizations. Each motif-pair ROC curve is obtained by applying the ROC analysis to the FCM generated probability of each network to belong to cluster one (see methods). **(B)** The resulting AUC as a function of sub-network size for motif 98 (red) is higher than for all possible pairs of motifs (black curves). The AUC shown is for comparing anti-correlated and correlated networks. **(C)** When the motif count is pooled across *N*_*av*_ = 20 realizations some two-motif curves exceed the single motif curve, which are shown separately in panel **(D)**. This suggests that for a specific size of the sub-network distinguishability can be improved by considering pairs of motif counts.

The question is whether this result can be improved by including counts for multiple different motifs (Figure 9A). Without pooling, motif 98 by itself outperforms any pair of motif counts, according to the AUC value (Figure 9B). To determine the AUC value for pairs of motif counts we used the FCM procedure as outlined in the methods section. When counts are pooled (Figure 9C), some motif pairs outperform motif 98 by a small margin. The pairs are highlighted in Figure 9D, and involve motif 98 itself. The more separated the cloud of points corresponding to different network types is, the better the FCM procedure classifies the networks, compare the plusses (correct discrimination) and dots (incorrect) in Figure 9A.

## Discussion

The overall firing rates in barrel cortex (de Kock et al., 2007; Greenberg et al., 2008; Barth and Poulet, 2012) are much lower than might be expected based on the classic experiments in macaque visual cortex (Hubel and Wiesel, 1968). Neural activity is also variable, which can be characterized as across trial reliability, or in terms of the coefficient of variation and Fano factor of spontaneous activity (Shadlen and Newsome, 1998). These measures reveal that the activity is similar to that of a Poisson process, in which the occurrence of a spike in a time bin is uncorrelated with whether or not a spike occurred in previous bins. The mean firing rate is maintained by the intrinsic excitability of neurons and their synaptic inputs, including recurrent excitation. High variability together with a low firing rate implies that the network dynamics should be stable against fluctuations in the mean activity in the sense that these fluctuations do not generate states with networks bursts in which all neurons in the network are active at the same time.

Experiments show that rodents can detect single-cell stimulation in barrel cortex, in which a single neuron is electrically stimulated to produce a high-frequency train of action potentials (Houweling and Brecht, 2008). This may mean that single-cell stimulation can cause an increase (or decrease) in the firing rate of the local network that is significantly different from that occurring during spontaneous activity. Taken together, this means that cortical networks with a low firing rate should at the same time be stable against fluctuations in firing rate and sensitive to weak stimulation. The overall goal of this paper was to a find a potential explanation for how the contrasting demands of sensitivity and stability can be realized. To achieve this we examined the dynamics of binary neural networks with correlation between the in- and out-degrees of neurons. In the following we summarize the main results with the aim of linking the detection performance of the network to experimentally obtained behavioral results, the mechanism by which sensitivity and stability can be achieved, and predicting the anatomical signatures of the hypothesized network. We also discuss the role of other biophysical factors, such as inhibition, not taken into account in the present study.

### Generating the network connectivity underlying enhanced stability

Our guiding hypothesis is that networks with an anti-correlation between the in- and out-degree of neurons are more stable and equally sensitive as other networks with comparable marginal degree distributions. Network sensitivity is generated by neurons with a high out-degree, because these would amplify the effect of nanostimulation the most. This amplification would also destabilize the network, so these cells should not be activated during spontaneous activity. As the input to neurons is proportional to the mean firing rate and their in-degree, this can be achieved by making sure that high out-degree neurons have low in-degrees. To maintain the average degree, both in and out, there then also need to be neurons with a low out-degree and a high in-degree. We implemented this hypothesis as an anti-correlation in the in- and out-degree.

In the standard Erdos-Renyi networks, the relative variance in the degree distribution for large networks becomes too small to have out-degrees that are much larger than the mean degree, which is needed to reach the desired sensitivity. Hence, we needed to broaden the degree distribution artificially by using a truncated bivariate Gaussian distribution. Networks with this sampled degree distribution were generated via the configuration model (Newman, 2010). This configuration model generates networks with self-edges and multi-edges. Analytical calculations show that the probability for obtaining a network with one or more of these edges is close to one for the large mean degrees we consider (Blitzstein and Diaconis, 2006). Nevertheless, the number of these edges is low and their impact on the dynamics was limited.

There are a number of ways to address the multi and self-edge problem in a more principled approach that differ in computational efficiency and ease of implementation. First, one can use the configuration model procedure, but reject an invalid edge and find a valid replacement. This carries the risk that the algorithm stops when there are no valid edges available, which means that the whole procedure has to be restarted. Alternatively, as mentioned before, one can identify the invalid edges when the network construction has been completed and remove them or replace them by valid ones. See Blitzstein and Diaconis (2006) for a review. Second, one can find one graph that satisfied the degree distribution using the Havel-Hakim procedure (Viger and Latapy, 2005; Erdos et al., 2010; Chatterjee et al., 2011) and generate samples from the overall graph distribution by swapping links (Blitzstein and Diaconis, 2006). Swapping links refers to the procedure where randomly chosen existing links *i* → *j* and *k* → *l* are swapped into *i* → *k* and *j* → *l* when this yields a simple graph without self-edges and multi-edges. This requires careful calibration of the number of swaps and also introduces bias because these swaps do not change the number of triangles in the network (Roberts and Coolen, 2012). Third, a sequential method can be defined that produces all possible graphs, by randomly selecting amongst the allowed edges that keep the residual degree distribution graphical (Del Genio et al., 2010; Kim et al., 2012). A degree distribution is graphical when there exists a simple graph with that distribution, after each step the degree distribution is lowered to account for the connections realized, and this is referred to as the residual degree distribution. This method does not produce the graphs with the correct probability. Hence, averages based on these graphs have to be reweighted to take this into account. Furthermore, in our hands, an implementation of this method produces graphs with a correlation between the in- and/or out-degrees between different nodes, which is referred to as assortativity. This necessitates a number of link swaps to remove these correlations. Fourth, edges can be sampled according to a Boltzman function (Park and Newman, 2004), where the expectation value of the degree of a node is fixed through a Lagrange multiplier, for which the appropriate value has to be picked, which can be achieved, for instance, through a maximum likelihood approach or iterative rescaling (Chatterjee et al., 2011). Taken together, we opted to use the simplest method here, because these alternative methods for network generation were computationally more intensive and also suffered from aforementioned additional drawbacks, such as graphs that were not sampled according to a uniform probability (Del Genio et al., 2010) or other biases in the network statistics (Roberts and Coolen, 2012). Recently developed methods for generating networks with degree correlations, both in a single neuron as well as between pairs of neurons look very promising (Roberts and Coolen, 2012).

### Stability is enhanced when the in- and out-degree are anti-correlated

Our aim was to find stable networks, by which we mean that fluctuations do not cause a cascade of recurrent excitation resulting in all cells being active at the same time. One solution would be to have inhibitory neurons, but this does not affect the stability of the LFS, it just changes the ultimate level of activity reached (Avermann et al., 2012). Stability can be assessed in a number of different ways. First, stability in the nonlinear system sense: is the LFS a fixed point of a noise-less, infinite size system? We determined that there was a range of coupling strengths *J*, below *J*_*c*_, for which such a LFS exists. The higher the baseline firing rate, the smaller that range is. Finite-size systems have a smaller range of stable coupling strength, because there is heterogeneity, not every neuron has the same in-degree. For instance, the uncorrelated network had a higher variance in the degree distribution than the ER network, and also had a smaller *J*_*c*_. Interestingly, networks with a positive correlation between in- and out-degrees reduced stability even more, leading to a lower *J*_*c*_, whereas for networks with a degree anti-correlation, *J*_*c*_ was higher, even exceeding the value for the ER network of the same size.

These calculations ignore the effects of fluctuations, which we subsequently introduced by making the dynamics stochastic. This did not alter the stability as determined before in terms of the existence of the LFS, but introduced other features. The LFS has a BOA with a fuzzy boundary due to the stochastic dynamics. A network can then be unstable when the fluctuations are large enough to leave the BOA when you wait long enough. This is primarily a concern for *J* values close to (and below) *J*_*c*_. We determined the fraction of trials during which the network left the LFS BOA during the simulated time interval. As expected the anti-correlated network is more stable, because *J*_*c*_ is larger. For coupling constants away from *J*_*c*_, this way of characterizing the BOA does not work. Hence, we started the network in states with many more neurons active than would be expected as a result of any normal fluctuation, and determined whether it converged to the LFS or HFS. This revealed that the BOA was larger for the anti-correlated network even away from *J*_*c*_.

Taken together, these results clearly show that anti-correlated networks are more stable than uncorrelated ones, which means they can operate stably at higher coupling strengths and baseline firing rates, which confers advantages when the sensitivity is higher for higher coupling strengths and baselines rates. Furthermore, their sensitivity is enhanced compared to ER networks with the same connection probability, because of a subset of neurons with a high out-degree.

Recent experiments summarized in Barth and Poulet (2012) show that the average firing rate in sensory cortex is low, especially in superficial layers. This holds for spontaneous as well as evoked activity, and for both anesthetized animals and awake animals and is the basis for the parameter settings in the model. Nevertheless, there is a small subset of cells that display high firing rates. Cells that have recently been active express the immediate-early gene *c-fos*. When the *c-fos* promoter is used to express the fluorescent marker GFP, the recently active cells can be targeted for recording *in vivo* and *in vitro*. The so called fosGFP+ cells had a higher firing rate both *in vivo* and *in vitro* and received more excitatory inputs and less inhibitory inputs (Yassin et al., 2010). Furthermore, these cells are more likely to be connected amongst themselves. In the anti-correlated networks, there are neurons with a high in-degree but a low out-degree which make the network more stable, and neurons with high out-degree but low in-degree that make the network more sensitive. The fosGFP+ neurons could correspond to the former group, which form the backbone for the spontaneous activity. We did not explicitly build in assortativity in the network to preferentially connect high in-degree neurons to each other as suggested by Yassin et al. (2010). We take from this result that the prevailing homeostatic processes create networks with more strongly connected sub-networks and produce cell-to-cell heterogeneity in the balance between excitation and inhibition. Training to detect electrical stimulation should thus be able to induce similar changes in network structure.

### The sensitivity estimated using different measures of network activity

Rodents were able to distinguish between patterns of neural activity during spontaneous activity and those caused by single-cell nanostimulation. Nevertheless, this distinction was small, given the effect size measured experimentally (Houweling and Brecht, 2008). One hypothesis is that the total amount of activity (firing rate) due to nanostimulation significantly exceeds that expected of a typical fluctuation. For a stationary network dynamics, this implies a fixed threshold above which a fluctuation is more likely caused by nanostimulation, whereas fluctuations below the threshold are more likely due to spontaneous activity. This can be quantified using a ROC curve, and the area under it, the AUC. The ROC is the curve traced out by varying this threshold and plotting the true positive rate (nanostimulation above threshold) vs. false positive (spontaneous fluctuations above threshold). When both distributions for the fluctuations are Gaussians, the AUC corresponds to the difference in means divided by the (common) standard deviation (Kingdom and Prins, 2010). Hence it is a measure of the difference in response relative to the size fluctuations around it. We found that the main determinant of the AUC is the out-degree of the stimulated neurons, independent of the correlation between in- and out-degree in the network. The AUC increases with coupling strength and baseline firing rate. The anti-correlated network has an advantage because it allows for a broader range of *J* and *r*_0_ values. It thus has an increased stability at equal sensitivity.

The above represents an underestimate of the sensitivity, because it assumes that the activity of each neuron contributes equally to the detection (decision) and that the temporal signature of the firing rate fluctuation is not informative. Our further analysis shows that each of these factors would improve detection performance and makes it therefore likely that state-of-the-art classification approaches such as support vector machines would even further improve performance. Taken together this means that as a system the rodent brain could reach a much higher sensitivity than predicted here, when it could utilize all the information available in the network activity. Model simulations of spike pattern detection by cortical networks (Haeusler and Maass, 2007) suggests that laminar models with plastic synapses allow for more accurate estimates of the detection capability compared to neural networks that do not take into account the layered structure of cortex.

### Detecting signatures of anti-correlated degree distributions

The model makes the prediction that anti-correlated networks would be more appropriate for the detection of nanostimulation in stable networks. To test this prediction we need to be able to distinguish correlations in the degree structure of the network without having access to all the inputs and all the outputs of a subset of neurons. We find that anti-correlations change the frequency of specific network motifs in a way that is independent of the network size, which means that it can be determined by averaging across many smaller sub-networks. A “ring” motif, number 98, which was a projection from neuron 1 to 2, from 2 to 3 and from 3 to 1, discriminated best between correlated and anti-correlated networks (Figure 7). Pairs of motif counts increased discriminability to a small extent, and only when the counts were pooled. This shows that these networks can be detected experimentally based on sampling sub-networks comprised of 30 neurons, when enough samples are available.

### Future studies should incorporate multiple types of interneurons

The model was highly simplified so that we could focus on the connectivity structure. Having established the advantages of anti-correlation, our goal is to study the effects in more realistic networks. There are many other biophysical features that could be included in the model that would change the results quantitatively or, in some cases, even qualitatively. Here we highlight a small selection of the most relevant ones.

The first issue is inhibition. Experimental evidence shows that two types of inhibitory neurons, those expressing parvalbumin (PV) and somatostatin (SOM), are relevant in determining the gain of the response of pyramidal cells to whisker stimulation, visual stimulation or current injection (Gentet et al., 2010; Kwan and Dan, 2012; Lee et al., 2012). Avermann and coworkers (Avermann et al., 2012) constructed a model of L2/3 in barrel cortex constrained by *in vitro* measurements and studied the effect of stimulating varying amounts of pyramidal cells expressing channelrhodopsin by light pulses. In this model the strongest projection, in terms of the connection probability and synaptic strength, was from pyramidal cells to fast spiking (FS) interneurons (corresponding to PV neurons). Even when a relatively small fraction of the pyramidal cells were stimulated, almost all FS cells were recruited. For higher fractions of stimulated pyramidal cells, the non-fast spiking (NFS) interneurons (such as SOM interneurons) would become gradually activated. As a result the pyramidal cell activity remained low despite strong stimulation. The authors hypothesize that the strong inhibition is a mechanism to maintain sparse spiking in the pyramidal cells, with the NFS cells providing a back-up inhibitory mechanism. It is not clear how this computational model would be applicable to *in vivo* dynamics where FS cells are already spontaneously active. Furthermore, the level of activity in the different interneurons depends on brain state (Gentet et al., 2010). We have simulated binary networks with inhibitory neurons and find that anti-correlated degree distributions in the E–E sub-network improve stability (and yield the same sensitivity).

Detection could also take place by a state change in the network. The network has a LFS and a not too biologically plausible HFS, which in the context of a network with inhibition would perhaps correspond to something like an upstate. The true positive rate would correspond to how often single-cell stimulation would drive the network out of the BOA for the LFS, whereas the false positive rate would correspond to how often this would happen in the spontaneous state. The latter is given by the fraction of trials the system goes to the HFS state (Figure 3). The former can be tuned by changing the number of neurons and the duration of stimulation. A proper examination of this issue would require a network with a population of inhibitory neurons (Avermann et al., 2012).

A second issue is the effect of including spike timing. Synapses are sensitive through short-term depression and facilitation to the temporal patterns of stimulation (Abbott and Regehr, 2004), which could thereby affect the postsynaptic response in a nonlinear fashion, thereby preferentially activating specific populations of neurons. Dendritic nonlinearities also affect the impact of synaptic inputs based on their temporal coincidence and whether they arrive on the same part of the dendrite (Gasparini et al., 2004; Major et al., 2008; Polsky et al., 2009; Lavzin et al., 2012). Either of these effects could increase the sensitivity to external stimulation, while not appreciably changing the stability, thereby strengthening the results reported in this paper. However, to fully quantify these effects would require new and more extensive simulations that fall outside the scope of this paper.

### Perceptual relevance of electrical or optical stimulation in experiment

Our study explores a hypothesis for how to achieve detection of an electrical stimulation by quick recurrent excitation that escapes before being shut down by inhibition, without destabilizing the spontaneous state. We now review the relevant literature focusing on the difference between electrical and sensory stimulation and the role of inhibition.

The barrel cortex normally processes thalamic activity generated in response to whisker stimulation. According to the canonical cortical circuit (Douglas and Martin, 2004; Lefort et al., 2009; Petersen and Crochet, 2013) this activity arrives first in layer 4 (L4) of the barrel column representing the stimulated whisker and then goes to L2/3 and subsequently to L5. It stands to reason that when during a task an animal needs to make a decision based on whisker stimulation, this is based on activity in L2/3 or L5 that came there by way of L4. The path taken by activity induced by optical, micro- or nanostimulation does not necessarily directly involve L4 and improving detection could thus require altering the underlying cortical circuit.

When monkeys were trained to detect microstimulation at a location in the visual cortex corresponding to a specific retinotopic location, the stimulation threshold for detection was reduced from about 50 μA to 5 μA over a few thousands of trials (Ni and Maunsell, 2010). At the same time the contrast threshold needed to detect real visual stimuli at the same retinotopic location increased from 4–8% to 8–60%. When the monkeys were subsequently retrained on detecting visual stimuli, the sensitivity was recovered in another few thousand trials, but the sensitivity to electrical stimulation was reduced. One possible interpretation is that learning to detect electrical stimulation reorganizes the recurrent circuits in L2/3 to become more sensitive at the expense of the L4 to L2/3 feedforward connection.

The animal improves its performance when learning to detect microstimulation, which could also be the case for single-cell nanostimulation modeled here. This improvement could occur because of one or more of the following reasons. First, the network could become more anti-correlated by changing the in-degrees. This means that the stability of the network would improve over time and perhaps that the number of false positives would reduce. Second, the out-degree of the stimulated neurons could increase, so that the nanostimulation signal becomes louder, hence the true positives should increase. Third, the neurons involved in the detection process become more sensitive to neurons directly downstream of the stimulated cells.

The threshold for detecting microstimulation in monkey visual cortex matches the strength necessary to elicit action potentials in mouse and cat cortex in the neighborhood of the electrode, 5–10 μA (Histed et al., 2009) and in rat barrel cortex 2–5 μA (Houweling and Brecht, 2008). These numbers did not depend on whether metal or glass pipette electrodes were used. Stimulation close above this threshold activated a set of widely dispersed neurons within a few hundred microns from the electrode, through antidromic action potentials in axons that are close to the electrode. As a result, the spatial pattern of activation was very sensitive to small changes in the location of the electrode.

Similar stimulus strength, 10 μA for 0.1 to 0.5 ms, yielding charge transfers on the order of 1 nC, applied in the infragranular layers could be detected in rats (Butovas and Schwarz, 2007). The authors (Butovas and Schwarz, 2003) estimate that this corresponds to activating 80% of the pyramidal cells within 450 micron of the electrode, yielding an increase in their firing rate of 25% corresponding to about 0.5 excess spike per neuron. Interestingly, trains of electrical stimulation were more effective, indicating that temporal correlation may be necessary to distinguish stimulation from spontaneous activity. Physiological measurements indicated that synapses of pyramidal cells on fast spiking interneurons depress more than the pyramidal to pyramidal synapses, which means that pulse trains could lead to more a prominent increase in activity than single stimuli (Holmgren et al., 2003).

Optogenetics was used to determine how many neurons in L2/3 would be required to generate a change in activity that would be detectable by a mouse (Huber et al., 2008). The authors' estimate of 300 neurons producing one action potential was based on a measured distribution of light intensity thresholds necessary to elicit an action potential, the number of neurons expressing the light-sensitive channelrhodopsin (ChR2) channels and the spatial fall off of the light intensity, and represents according to these authors an overestimate. The number of 300 neurons corresponds to about 5% of the approximately 6500 neurons present in a mouse barrel column (Lefort et al., 2009).

Nanostimulation refers to electrical activation of an individual neuron with a glass pipette in the juxtacellular configuration. Nanostimulation in rat barrel cortex must have led to behaviorally relevant changes in network activity, as the animal was able to detect nanostimulation, but the average effect size was rather small (Houweling and Brecht, 2008). The nature of this activity could not be assessed, but experiments in mouse visual cortex may shed some light on this. Single-cell stimulation led to spikes in the stimulated neuron and calcium transients in some of the surrounding neurons that could be detected using two-photon microscopy (Kwan and Dan, 2012). Such stimulation induced postsynaptic activity in very few other pyramidal cells, 20 out of 1152 measured. SOM interneurons [corresponding to the NFS of Avermann et al. (2012)] were most strongly activated, 5 out of 17 measured. PV expressing cells did not respond to this stimulation, but their calcium transients were most strongly correlated to the network activity produced by the rest of the measured cells. This indicates that in this state the SOM cells would be required to damp the increase in activity generated by the recurrently connected pyramidal cell network.

## Summary

Taken together, experimental results suggest that detection of single-cell stimulation requires a quick propagation of excitatory cell activity, before the various types of inhibition kick in. Our studies indicate that anti-correlated degree distributions could be an important strategy for increasing sensitivity while maintaining stability.

## Author contributions

Paul Tiesinga and Arthur R. Houweling designed the research project, Paul Tiesinga and Juan C. Vasquez wrote the code, Juan C. Vasquez and Paul Tiesinga performed the simulations, Paul Tiesinga wrote the manuscript together with Arthur R. Houweling.

### Conflict of interest statement

The authors declare that the research was conducted in the absence of any commercial or financial relationships that could be construed as a potential conflict of interest.
